# Prise en charge du phéochromocytome bilatéral - à propos d'un cas

**DOI:** 10.11604/pamj.2014.18.97.1916

**Published:** 2014-05-27

**Authors:** Khalid Khattala, Aziz Elmadi, Abdelhalim Mahmoudi, Mohamed Rami, Youssef Bouabdallah

**Affiliations:** 1 Service De Chirurgie Pédiatrique Chu Hassan II De Fès, Maroc

**Keywords:** Phéochromocytome bilatérale, hypertension arterielle, réanimation, surrénalectomie partielle, Management of bilateral pheochromocytoma - about a case, high blood pressure, resuscitation, partial adrenalectomy

## Abstract

Le phéochromocytome est une pathologie rare et sévère; sa survenue à un âge précoce, sa bilatéralité et son association à des signes de gravité en font la sévérité. Notre observation en fait l'exemple; le phéochromocytome bilatéral est révélé par un accident vasculaire ischémique qui a dominé le tableau initial chez une fille de 10 ans. L'imagerie a orienté vers le diagnostic (échographie et TDM abdominales), confirmé par le dosage des catécholamines urinaires. Après une préparation soigneuse, l'exérèse chirurgicale (surrénalectomie totale droite et partielle gauche), avec bonne surveillance péri opératoire, ont permis d'avoir de bons résultats. L’étude histologique a étayé la nature anatomo pathologique de la pièce opératoire.

## Introduction

Le phéochromocytome est une tumeur rare développée au dépend des cellules chromaffines. Chez l'enfant, cette tumeur pose un problème de diagnostic, compte tenu de sa rareté et des différents signes cliniques associés. La confirmation du diagnostic repose sur les dosages des catécholamines urinaires ou plasmatiques, de leurs précurseurs ou de leurs métabolites. Le diagnostic topographique a bénéficié du développement de l'imagerie, notamment de l'IRM et de la scintigraphie à la méta-iodobenzylguanidine (MIBG). La prise en charge thérapeutique est multi disciplinaire. L’étude anatomopathologique de la pièce opératoire apporte la confirmation histologique du diagnostic. Nous rapportons le cas d'une fille de 10 ans, qui présente un phéochromocytome bilatéral révélé par un accident vasculaire cérébral ischémique (AVC) qui a bien évolué après traitement chirurgicale, nous allons aborder dans ce travail le chapitre de la prise en charge des formes bilatérales, avec l’évolution des techniques et des difficultés.

## Patient et observation

Fille de 10 ans, sans antécédents particuliers, admise pour trouble de conscience, qui présente le jour même de son admission des céphalées en casque, associées à des vomissements en jet, des sueurs profuses, évoluant dans un contexte de fièvre non chiffrée. Avec installation 5 heures après d'un déficit hemicorporel droit. L'examen trouve un enfant fébrile à 40°;C, normotendue, eupnéique à 3O cycles par minute, une fréquence cardiaque à 100 battements par minutes, un poids de 25kg et une taille de 130cm (moins une déviation standard).

L'examen neurologique trouve un enfant obnubilé, sans raideur de la nuque. Les Signes de Koernig et Brudzinski sont négatifs. Elle présente une hémiplégie flasque avec une hémianesthésie droite. La TDM cérébrale initiale montre une plage hypodense mal systématisée au niveau temporo pariétal gauche. Une ponction lombaire ramène du liquide clair avec 25 éléments blancs/mm3, une glycorachie et une proteïnorachie normales. L'examen direct est négatif. L'hémogramme montre une hémoglobine (Hb) à 13,5g/dl, un taux de plaquettes à: 447000 éléments/mm3, et des globules blancs à: 12300/mm3. La vitesse de sédimentation (VS) est de 75mm à la première heure, 135mm à la deuxième heure. La CRP est à 136 mg/l. L'ionogramme sanguin normal.

**Figure 1:**
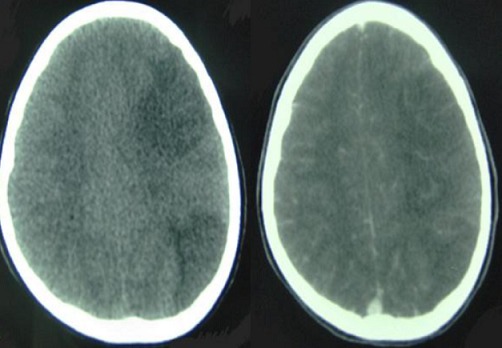
Image tomodensitométrique cérébrale objectivant un AVC ischémique systématisé du territoire de l'artère sylvienne superficielle gauche. A gauche: Sans injection de produit de contraste; A droite: Après injection du produit de contraste

Après 2 jours de traitement anti viral comme meningoencephalite herpétique, l’évolution est marquée par la détérioration de l’état neurologique, ce qui a nécessité une intubation avec ventilation artificielle. A son 5ème jour de traitement, une TDM cérébrale refaite est revenue en faveur d'un accident vasculaire ischémique (AVC) systématisé du territoire de l'artère sylvienne superficielle gauche (Figure 1). L'enfant est mise sous traitement anticoagulant, anti inflammatoire devant la suspicion d'un AVC ischémique secondaire à une vascularite. Parallèlement, on a poussé les investigations en vue d'un diagnostic étiologique qui est revenu négatif.

L’échocardiographie met en évidence une hypertrophie ventriculaire gauche et une dilatation en fuseau des artères coronaires. L'ECG montre un remaniement électrique en septo apical. Une troponine Ic demandée en urgence est revenue négative. L’évolution, sous traitement anticoagulant et anti inflammatoire, est marquée par un début de récupération sur le plan neurologique. Par ailleurs, à son 20ème jour après le début de la symptomatologie, elle a présenté des pics hypertensifs arrivant jusqu’à 160/100mmHg. L'enfant est mise sous traitement médical anti hypertenseur à base de Béta bloqueur (Propranolol Avlocardyl^®^ à dose de 50mg/m^2^/jour, et inhibiteur de l'enzyme de conversion (IEC) (Captopril Lopril^®^ à dose de 1mg/kg/jour).

**Figure 2:**
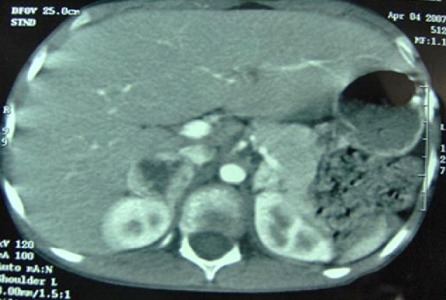
TDM abdominale: masse surrénalienne droite faisant32/24mm, et gauche faisant 20mm

Le bilan de l'HTA, confirmée cliniquement est complété par l’échographie abdominale qui met en évidence la présence d'une lésion tissulaire surrénalienne droite hypo échogène hétérogène, bien limitée, à contours réguliers, non vascularisée au doppler couleur, mesurant 46 sur 33mm de grand diamètre au niveau du pôle supérieur du rein droit, et une structure tissulaire arrondie échogène vascularisée, de 17mm de grand axe en regard du pôle supérieur du rein gauche. Par ailleurs, les reins sont normaux, et le doppler des artères rénales ne note pas d'anomalie morphologique ou hémodynamique. La TDM abdominale (Figure 2), objective la présence d'une lésion surrénalienne droite tissulaire, qui se rehausse de façon intense et hétérogène après injection de produit de contraste présentant une nécrose centrale, cette lésion est bien limitée, ses contours sont réguliers, mesurant 32 sur 24mm de diamètre, n'infiltrant pas les structures avoisinantes; la présence également d'un autre nodule tissulaire gauche, hypodense qui se rehausse de façon intense et homogène par le produit de contraste, arrondi, bien limité, de contours réguliers, mesurant 20mm de grand axe, sans signes d'extension aux organes de voisinage. Un phéochromocytome bilatéral est fortement suspecté. Le dosage des catécholamines urinaires fractionnées sur urines de 24h montre une dopamine de 2,97micro mol/24h (la normale est inférieure à 0,5micro mol/24h). Une étude génétique à la recherche d'un caractère familial de la maladie est normale.

La malade a été opéré par une incision médiane à cheval sur l'ombilic, abord transperitoneal, l'exploration trouve une masse tumorale faisant 5cm sur 3cm au dépend de la surrénale droite; ligature et section du pédicule surrénalien supérieur, puis surrénalectomie totale droite. Alors qu’à gauche, on trouve une masse faisant 1cm de grand axe, avec tissu jaune chamois faisant rappeler le parenchyme surrénalien. Une surrénalectomie partielle gauche est effectuée.

**Figure 3:**
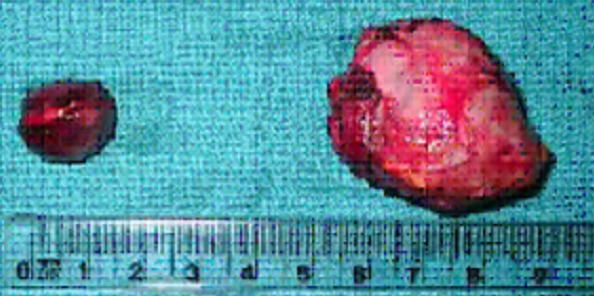
Aspect macroscopique des pièces opératoires: Les 2 pièces sont homogènes, bien limitées. A droite, pièce faisant 4cm de grand axe, à gauche, pièce faisant 2cm de grand axe

**Figure 4:**
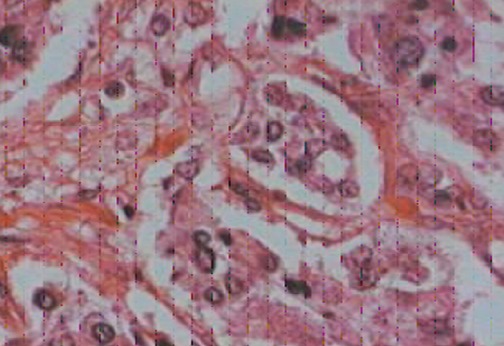
Aspect microscopique: grossissement x200: Prolifération tumorale en cordons faite de cellules monomorphes avec un stroma vasculaire

Les pièces opératoires sont adressées à l'anatomopathologie pour étude histologique qui a confirmé le diagnostic (Figure 3, Figure 4).

En post opératoire, la patiente est admise au service de réanimation, pour qu'elle soit extubée après 6 heures, après stabilisation respiratoire et réveil complet. Ses suites opératoires immédiates sont simples en dehors d'une hypotension passagère qu'elle a présentée et qui a été jugulée par sa mise sous adrénaline. L'hormonothérapie substitutive à base d'hydrocortisone par voie orale (10mg/m^2^/jour) est maintenue pendant 1 mois, jusqu’à la réalisation d'un test au synachtène^®^ objectivant chez elle une réponse normale du parenchyme surrénalien restant, justifiant la dégression progressive de l'hydrocortisone.

Un Holter tensionnel de 24h fait à son 45ème jour du post opératoire, affirme la normalité des chiffres tensionnels. Par ailleurs, des échocardiographies à répétition ont objectivé la régression progressive du volume du ventricule gauche, et du diamètre des artères coronaires au bout de 2 mois.

Notre patiente est sortie, après 2 mois du post opératoire, avec des chiffres tensionnels normaux et une fonction surrénalienne bien évaluée sans traitement substitutif. Durant la consultation bi hebdomadaire, la fille est bien contrôlée, commençant à récupérer son déficit moteur, devenant de plus en plus autonome. Devenue normale après un recul de 5 ans.

## Discussion

Décrits en 1886 par Frankel, les phéochromocytomes ont fait l'objet des premières exérèses en 1926 par Mayo et Rox [1]. En 1951, une revue de la littérature a relevé 25% de décès péri opératoires en relation avec les variations tensionnelles à type d'hyper ou d'hypotension [2]. 50 ans plus tard, Kvale et al. publient la première série de 51 patients opérés pour phéochromocytome sans décès, grâce à l'utilisation de phentalamine et norépinephrine [3]. Dès lors, il fut classique de proposer, avant l'intervention, un blocage progressif des récepteurs alpha sur une à deux semaines, pour diminuer l’état d'hyperadrénérgie, et restaurer la volémie. D'abord il faut faire une préparation dont les objectifs furent précisés par Roïzen et al. en 1978 [4]: « normaliser la tension artérielle et les répercussions electrocardiographiques des cardiopathies sous jacentes le plus souvent hypertrophiques, tout en amenant le patient sur le versant des effets secondaires, hypotension orthostatique et congestion nasale ».

Les modalités diffèrent selon les équipes [5]. L'utilisation des médicaments bloquant les récepteurs alpha adrénergiques vise à corriger l'HTA et en prévenir les poussées paroxystiques. Ces alpha bloquants permettent également de normaliser la volémie quand celle-ci est diminuée, et de sensibiliser à nouveau les récepteurs alpha [5], ils ont modifié profondément le pronostic opératoire du phéochromocytome.

Depuis les années 1980, les inhibiteurs calciques de la famille des dihydropyridines se sont avérés efficaces dans la préparation des phéochromocytomes à la chirurgie [6, 7]. Ils présentent peu d'effets secondaires.

Notre patiente a été mise sous béta bloqueurs et IEC, après 3 semaines, on’ est arrivé à stabiliser ses chiffres tensionnels.

La règle d'or de la chirurgie du phéochromocytome est de “ Disséquer doucement le patient de la tumeur, et non la tumeur du patient” [8].

Les voies chirurgicales d'accès comme la voie transpéritonéale antérieure, la voie postérieure ou la lombotomie sont des voies d'abord difficiles, associées à un taux élevé de mortalité et à une convalescence prolongée [9]. Les complications des surrenalectomies par voie ouverte sont plus souvent en rapport avec ces difficultés d'accès qu'avec les exérèses glandulaires [10]. Depuis les premiers cas de surrénalectomies laparoscopiques rapportés par Gagner en 1999]. Au début, le consensus général était de réserver l'abord vidéo endoscopique aux tumeurs de moins de 6cm sans tenir compte du risque de malignité [10], il est maintenant démontré qu'au sein d'une équipe entraînée, les surrénalectomies vidéo endoscopiques sont réalisables sans risque particulier pour les tumeurs de plus de 6cm, mais cela ne paraît pas modifier radicalement les résultats de l'exérèse [10]. Deux voies d'abord sont possibles dans la chirurgie endoscopique: la voie transpéritonéale et la voie rétro péritonéale.

Sur le plan anatomopathologique, les phéochromocytomes sont caractérisés par un important polymorphisme architectural d'une zone à une autre. Ils sont constitués de phéochromocytes organisés en formations trabéculaires ou rubanées. Le diagnostic histologique est étayé par la mise en évidence, dans les cellules tumorales, de granulations argyrophiles par la coloration de Grimelius et par des techniques d'immunohistochimie [6].

L’évolution du phéochromocytome non traité est toujours grave, souvent mortelle quant aux phéochromocytomes opérés, à cours terme, leur pronostic est aujourd'hui très bon dans la majorité des cas, la mortalité péri opératoire ayant énormément diminué [6]. Les difficultés sont surtout liées au diagnostic des récidives; en effet, une récidive peut survenir de nombreuses années après le diagnostic initial, les écarts allant de 1 à plus de 20 ans chez les adultes [6].

## Conclusion

Le phéochromocytome est une pathologie rare et sévère; sa survenue à un âge précoce, sa bilatéralité et son association à des signes de gravité en font la sévérité. Nous soulignons l'intérêt de la bonne collaboration multi disciplinaire entre pédiatres, radiologues, anesthésistes-réanimateurs, chirurgiens pédiatres, anatomo-pathologistes et généticiens, dans la prise en charge d'une telle pathologie comme le phéochromocytome bilatéral, aboutissant à un tel succès thérapeutique.

## References

[1] Lacoste L (2005). Préparation et environnement péri opératoire dans la chirurgie du phéochromocytome. Annales de chirurgie..

[2] Graham JB (1951). Pheochromocytoma and hypertension: An analysis of 207 cases. Int Abstracts surg..

[3] Kvale WF, Roth GM, Manger WM (1956). Pheochromocytoma. Circulation..

[4] Roizen MF, Hunt TK, Beaupre PN (1983). The effect of alpha adrenergic blockade on cardiac performance and tissue oxygen delivery during excision of pheochromocytoma. Surgery..

[5] Tavernier B Anesthésie Réanimation dans la chirurgie des surrénales. http://www.em-consulte.com/article/11582/anesth%C3%A9sie-r%C3%A9animation-dans-la-chiru.

[6] Dubois R, Chappuis JP (1997). Le phéochromocytome: Particularités pédiatriques. Archives de Pédiatrie..

[7] Serfas D, Shoback DM, Lorell BH (1983). Pheochromocytoma and hypertrophic cardiomyopathy: apparent suppression of symptoms and noradrenaline secretion by calcium-chanel blockade. Lancet..

[8] Zerhouni H, Kaddouri N, Abdelhak N, Barahioui M (2002). Le phéochromocytome de l'enfant, à propos de deux cas. Ann Urol..

[9] Redouane Rabii, Laurent Salomon, Fabien Saint (2001). Traitement des pheochromocytomes par laparoscopie retropéritonéale. Progrès en urologie.

[10] Henry JF, Sebag F (2002). Leçons retenues après 274 surrénalectomies laparoscopiques. Annales de chirurgie..

